# Use of Oral N-Acetylcysteine (NAC) in Non-Acetaminophen-Induced Acute Hepatic Failure

**DOI:** 10.7759/cureus.35852

**Published:** 2023-03-07

**Authors:** Saleem Sharieff, Asim Idrees, Wajid Rafai, Syed Uzair S Bukhari

**Affiliations:** 1 Critical Care Medicine, Pakistan Kidney and Liver Institute and Research Center, Lahore, PAK; 2 Medicine/Critical Care Medicine, Grand River Hospital, Kitchener, CAN; 3 Pharmacology, Pakistan Kidney and Liver Institute and Research Center, Lahore, PAK

**Keywords:** survival, hepatic encephalopathy, fulminant liver failure, oral n-acetylcysteine, acute liver failure

## Abstract

Background: Acute liver failure (ALF) is a syndrome rather than a specific disease with several possible causes, and viral hepatitis is a major cause. The objective of the study was to assess the benefit of N-acetylcysteine (NAC) in non-acetaminophen-induced acute liver failure (NAI-ALF).

Methods: A total of six patients with a diagnosis of acute liver failure (ALF) were included in the study. All six patients received oral NAC for 72 hrs. The parameters evaluated were demographic, clinical, biochemical, outcome, and length of ICU and hospital stay. The primary outcome was a reduction in mortality with the use of NAC in NAI-ALF. The secondary outcomes were to evaluate the safety of NAC and assess factors predicting mortality.

Results: All patients improved and returned to normal or near-normal liver function with the use of NAC. No side effects were noted, and the use of NAC was associated with a shorter hospital stay.

Conclusion: In patients with non-acetaminophen-related acute liver failure, N-acetyl-L-cysteine (NAC) significantly improves overall survival and also decreases the length of hospital stay.

## Introduction

Acute liver failure (ALF) is an uncommon but severe, life-threatening emergency causing rapid deterioration of liver function, leading to coagulopathy and encephalopathy in a previously healthy liver. The incidence of ALF is quite high, ranging between 2000 and 3000 cases per year in the US, while reported mortality is as high as 30% [1].

At present, acetaminophen is the foremost cause of ALF in the western world, accounting for nearly 50% of all ALF cases in the USA and 60% in the United Kingdom [2,3]. It accounts for up to 7% of all liver-related deaths and 6% of liver transplants [4,5]. Before transplantation, most series suggested a less than 15% survival rate in patients with acute liver failure (ALF). The overall short-term survival (one year), including those undergoing transplantation, is greater than 65% [4,5]. 

The common etiologies of ALF include viral hepatitis, drugs, and toxins, as well as autoimmune hepatitis [4]. In the USA, the commonest etiology for ALF is acetaminophen-induced, followed by other drug-induced ALF and viral hepatitis like HBV [1]. However, in the Indian subcontinent, viral hepatitis alone accounts for 90% of ALF [6]. 

Medical management includes supportive measures and treatment of the underlying cause. Although liver transplantation is a life-saving treatment, the availability of organs has always been challenging; therefore, other treatment options have been sought.

The use of N-acetyl-l-cysteine (NAC) as an antidote for acetaminophen toxicity causing acute liver failure was first reported in 1974, and since then, it is considered a standard of care in this fatal condition [7-11].

N-acetyl-l-cysteine (NAC) acts mainly on the supply of cysteine for glutathione (GSH) synthesis, thereby replenishing intracellular glutathione and exerting antioxidant effects that help to ameliorate the adverse consequences of the hepatic insult and its sequelae [10-12]. It also has a vasodilatory and inotropic affect. Thus, improving perfusion and oxygenation to vital organs during shock [10,13].

In 2011, the American Association for the Study of Liver Diseases (AASLD) guidelines suggested NAC may also be helpful in non-acetaminophen-related liver injury [14], both post-transplant and transplant-free survival, and decreasing the overall length of hospital stay [15-17].

There is not much data on the use of an oral form of NAC in ALF patients; therefore, we decided to use NAC in our non-acetaminophen-induced acute liver failure (NAI-ALF) patients. Also, most of the literature is on the use of IV NAC, and only a few reports are on the role of oral NAC in ALF patients. We at our center used PO NAC in ALF patients. 

## Materials and methods

Acute liver failure (ALF), also known as fulminant hepatic failure (FHF), is defined as the development of severe acute liver injury with impaired synthetic function (INR of ≥1.5) and altered mental status (encephalopathy) in a patient without cirrhosis or pre-existing liver disease. A commonly used cut-off to define acute liver failure is an illness duration of <26 weeks [18].

A total of six patients were admitted to our ICU at the Pakistan Kidney and Liver Institute and Research Centre (PKLI-RC), Lahore, Pakistan, with the diagnosis of acute liver failure (ALF) not related to acetaminophen toxicity between January 1st and December 31st, 2022. None of them had chronic liver disease (CLD). 

All were treated as per standard management for ALF, including antibiotics where necessary in accordance with the ICU protocol and culture sensitivity. N-acetylcysteine (NAC) was given from day one of ICU admission while the workup started for possible liver transplantation in case of no improvement or deterioration. The dosage used in our patients was 140 mg/kg, followed by 70 mg/kg, for a total of 17 doses, four hours apart, starting within 6 h of admission for a minimum of three days [10,15,16]. The major reason we opted for oral NAC was the unavailability of its IV formulations in Pakistan.

The objective of this study was to evaluate the effectiveness of early use of NAC in patients with acute fulminant hepatic failure secondary to non-acetaminophen-induced liver failure. The primary outcome was defined as survival with the improvement of symptoms and liver function vs. the requirement of a liver transplant or death.

Ethical approval was obtained from the ethics committee and the Institutional Review Board of the Pakistan Kidney and Liver Institute and Research Center, Lahore, Pakistan. (Approval number: PKLI-IRB/AB/103).

A brief summary of each case is given below:

Case no. 1

A 16-year-old previously healthy male presented with fever, diarrhea, vomiting, abdominal pain, an altered level of consciousness, and jaundice. He was referred to us by a community hospital. The initial workup was suggestive of acute liver failure (ALF), and the CT brain was negative for any acute abnormality. He was shifted to the ICU due to grade 4 encephalopathy and was electively intubated to protect the airway; he also required vasopressors. He was started on oral N-acetylcysteine. Serology was positive for Hepatitis E IgM and Dengue IgM. He received 72-hour oral NAC according to the protocol. His labs and encephalopathy improved gradually, and he was successfully extubated after four days of treatment and mechanical ventilation. He remained in the ICU for a total of seven days, after which he was transferred to the inpatient department for two more days and was then discharged home.

Case no. 2

A 21-year-old female with no known comorbid condition presented with a one-week history of fever followed by irritability, restlessness, nausea, vomiting, and jaundice for seven days. On arrival at our hospital, she was in grade 4 of hepatic encephalopathy secondary to acute liver failure. She was admitted to the ICU and intubated for airway protection. She didn’t require any vasopressor support. The CT scan was negative for any cerebral edema. She was started on oral NAC for 72 hours. Her ALF workup was positive for Hepatitis A IgM, Dengue IgM, and ANA (Titer 1:80). Her clinical and biochemical picture improved over subsequent days. She was successfully extubated on day two of mechanical ventilation. She remained in the ICU for three days, after which she was discharged to the inpatient department, where she stayed for another five days before going home.

Case no. 3

A 16-year-old male with no comorbidity was admitted with fever and jaundice for one week, followed by irritability and drowsiness. On arrival, he was in grade 4 hepatic encephalopathy and was therefore admitted to our ICU and intubated. He was diagnosed with acute liver failure. He didn’t require any vasopressor support, the CT brain was negative for cerebral edema or bleeding, and the serology was positive for hepatitis A IgM. He received 72 hours of oral NAC, according to the protocol. His mental status was not improving with high ammonia levels, so he underwent SLED for hyperammonemia. Gradually, his condition improved, and he was successfully extubated on day six of mechanical ventilation. He remained in the ICU for nine days, after which he was transferred to the inpatient department. He stayed there for four more days and was then discharged to go home.

Case no. 4

A 20-year-old female patient was referred from a primary care center for an altered level of consciousness. She had a history of fever, abdominal pain, jaundice, and drowsiness for the last seven days and was in grade 4 hepatic encephalopathy on arrival, requiring elective intubation and mechanical ventilation. She was hemodynamically stable, and the CT brain scan showed mild cerebral edema. She was started on 3% hypertonic saline and oral NAC. The serology was positive for hepatitis E IgM. She gradually improved and became alert and oriented. She was successfully extubated on day five of mechanical ventilation, and after a total of seven days in the ICU, she was transferred to the inpatient ward, where, after another three days, she was discharged home.

Case no. 5

A 24-year-old female was admitted to our ICU with a five-day history of feeling unwell, followed by jaundice and grade 4 hepatic encephalopathy. She was electively intubated, but as she was hemodynamically stable, she didn’t require any vasopressors. Her workup for ALF was positive for HBsAg and ANA (Titer 1:40). The CT brain was negative for cerebral edema or bleeding. She was also started on oral NAC for 72 hours. Due to hyperammonemia and low GCS, she underwent three sessions of SLED during her ICU stay. She gradually improved over the next four days and got extubated. After a total of seven days in the ICU, she was transferred to the inpatient ward, from which she was discharged home after another seven days.

Case no. 6

A 25-year-old female with no prior comorbid conditions was admitted with a five-day history of fever, vomiting, and jaundice, followed by altered mental status. She was diagnosed with acute liver failure, and serology confirmed hepatitis E IgM. She did not require vasopressor support, neither she required mechanical ventilation. She was given 3% saline since her CT brain suggested mild cerebral edema. She was started on oral NAC for 72 hours as per protocol. She showed dramatic improvement and became asymptomatic. He was transferred to the inpatient ward after a two-day stay in the ICU, where she completed the course of NAC. In the next six days, she was discharged home in stable condition.

## Results

From January until December 2022, six patients fulfilled the diagnosis of acute liver failure. Table 1 shows patient data, including demographic information, clinical and biochemical information, duration of illness, treatment received, and outcome. Viral hepatitis was the main etiology in all patients based on compatible clinical and laboratory findings, including positive serology. The median age was 20.5 years; there were two males and four females; three patients had HEV, two had HAV, and one had HBsAg, while two patients had co-infections with dengue fever. The most common presenting symptom (in all patients) was decreased level of consciousness from hepatic encephalopathy grade 4, and none of them had renal failure.

**Table 1:** Characteristics of patients with acute liver failure receiving N-acetylcysteine in non-acetaminophen liver failure

Variables (Reference range)	Case 1	Case 2	Case 3	Case 4	Case 5	Case 6	Reference Range
Age (years)	16	21	16	20	24	25	
Gender	Male	Female	Male	Female	Female	Female	
Total Bilirubin (0.2-1.2 mg/dL) (On admission)	6.6	6	11	6.6	7.1	3.3	0.2-1.2 mg/dL
Total Bilirubin (0.2-1.2 mg/dL) (Final)	7.4	6.1	12.2	3.9	2.8	3.6	0.2-1.2 mg/dL
ALT (0-55 U/L) (On admission)	2220	2788	1668	647	4065	1826	0-55 U/L
ALT (Final) ( 0-55 U/L)	330	244	87	77	162	248	0-55 U/L
AST (5-34 U/L) (On admission)	1124	1290	532	938	1250	419	5-34 U/L
AST (5-34 U/L) (Final)	82	49	61	65	60	54	5-34 U/L
INR (0.7-1.5) (On admission)	2.8	6.2	3.6	3.5	3.2	3.4	0.7-1.5
Lactate (0.2-1.8 mmol/L) (On Admission)	2.6	4.3	3.7 / 4.8	1.1	2.5 5.8	2.1	0.2-1.8 mmol/L
pH (7.35-7.45) (On Admission)	7.5	7.39	7.38	7.42	7.43	7.42	7.35-7.45
INR (0.7-1.5) (Final)	1.0	1.0	0.9	1.0	1.1	1.0	0.7-1.5
Hepatic Encephalopathy grade (On admission)	4	4	4	4	4	4	
Hepatic Encephalopathy (Final)	0	0	0	0	0	0	
Ammonia (31-123 µg/dL) (On admission)	185	142	163	363	239	106	31-123 µg/dL
Last Ammonia (31-123 µg/dL)	90	22	33	61	40	116	31-123 µg/dL
CT Brain showing cerebral edema	No	No	No	Yes	No	Yes	
Number of days in ICU/ward	7 / 2	3 / 5	9 / 4	7 / 3	7 / 7	2 / 6	
Comorbidity	None	None	None	None	None	None	
Etiology of ALF (Positive Markers)	HEV IgM, Dengue IgM	HAV IgM, Dengue IgM, Anti-HCV	HAV IgM	HEV IgM	HBsAg, Anti-HDV,	HEV IgM	
Recovery	Yes	Yes	Yes	Yes	Yes	Yes	

During the illness, five patients required mechanical ventilation. Two patients were offered sustained low-efficiency dialysis (SLED) for hyper-ammonia with a decreased level of consciousness (in one patient, ammonia increased from 163 to 383 while the other had 363 on admission).

The mean number of days of hospital admission was 10.33 ± 2.36, and all patients recovered completely, with the return of their LFTs to or near normal and no residual symptoms at the time of discharge. None of them required liver transplantation. On 3 to 6 months of telephonic follow-up, all of them were symptom-free and doing well.

## Discussion

Acute liver failure (ALF) is characterized by the acute onset of liver-based coagulopathy and biochemical evidence of hepatocellular injury resulting from rapid deterioration in liver cell function. In our study, there were six patients diagnosed with ALF who were offered oral NAC. All of them recovered completely without the need for liver transplantation and had a shorter hospital stay.

Previous studies have also shown improvement with NAC in NAI-ALF and transplant-free survival when used in early-stage non-acetaminophen ALF (both in adults and children) [17,19]. Studies by Mumtaz et al. [10] used oral NAC, while Nabi et al. [20] used IV NAC, and both studies showed improvement when NAC was used in ALF.

However, there was a study [5] where NAC was used in indeterminate ALF (excluding drug-induced ALF, viral hepatitis, autoimmune ALF, acute on chronic liver failure, ALF during pregnancy, and hepatic shock). The results did not show improvement in overall survival with NAC administration nor a reduction in the duration of hospital stay. However, this area needs to be further explored.

In our study, patients had viral hepatitis as a cause of ALF and showed recovery. Nabi et al. [5] found that advanced grade of encephalopathy (grades III and IV) and subacute presentation showed poor prognosis with age >50 years, III-IV grade of encephalopathy, renal impairment, MELD score > 30, and bilirubin > 20mg/dl was the independent prognostic factors determining mortality.

Our patients were younger and had signs of poor prognosis like high bilirubin, grade 4 hepatic encephalopathy, and the requirement for mechanical ventilation. These are comparable findings to other reports [10,21,22]. Khuroo et al. [21] reported mortality without liver transplantation at 72.8%, in which only supportive treatment was offered, while the use of NAC in our patients showed full recovery without the need for transplantation.

‌Oxidative stress (OS)

Free radicals, including reactive oxygen species, are molecules with one or more unpaired electrons. These include superoxide, hydroxyl radicals, and nitric oxide radicals. They are produced as a result of metabolic process within mitochondria. External substances, such as cigarette smoke, pesticides, and ozone, can also cause the formation of free radicals in the body. Long-term oxidative stress damages the body’s cells, proteins, and DNA.

The main function of antioxidants is to fight against oxidative stress (OS) through neutralizing or removing free radicals by donating an electron. Among these antioxidant systems, glutathione (GSH), which is a thiol antioxidant, protects cells against toxicity [10-12]. Oxidative stress (OS) is a state that occurs when there is an excess of free radicals in the body’s cells due to low levels of antioxidants, causing an imbalance of free radicals and antioxidants in the body and leading to cell and tissue damage. 

The antioxidant function of the liver

Reactive oxygen species (ROS) released by Kupffer cells (KCs) activate the hepatic stellate cells (HSCs), leading to an increase in proliferation and synthesis of extracellular matrix (ECM), which causes liver injury and leads to fibrosis and cirrhosis [23-25]. ROS and RNS (reactive oxygen and nitrogen species) can be removed in the liver by antioxidants like glutathione (GSH), vitamins C, A, and E, and enzymes such as superoxide dismutase (SOD), catalase (CAT), glutathione peroxidase (GPx), and thioredoxin [26].

N-acetylcysteine (NAC)

Acetaminophen overdose causing acute liver injury was first recognized in the 1960s [7]. Acetaminophen, which is metabolized primarily by sulfation and glucuronidation, can also be oxidized by CYPs to form N-acetyl-p-benzoquinone imine (NAPQI). NAPQI can then interact with cellular macromolecules, resulting in toxicity, or it can be detoxified by undergoing GSH conjugation. The role of acetylcysteine (NAC) as an antidote for acetaminophen toxicity was first recognized in 1974 [7-11]. Acetylcysteine (NAC) is a thiol-containing derivative of the amino acid cysteine, which is the acetylated precursor of reduced glutathione (GSH). The antioxidant activity of NAC may have both direct (the free sulfhydryl group may serve as a ready source of reducing equivalents) and indirect (through replenishment of intracellular GSH levels) antioxidant effects. This neutralizes N-acetyl-p-benzoquinone imine (NAPQI), which is responsible for hepatocyte toxicity. Thus, NAC may break the vicious oxidative cycle by reducing oxidative stress and, subsequently, inflammation [10-12]. 

**Figure 1:**
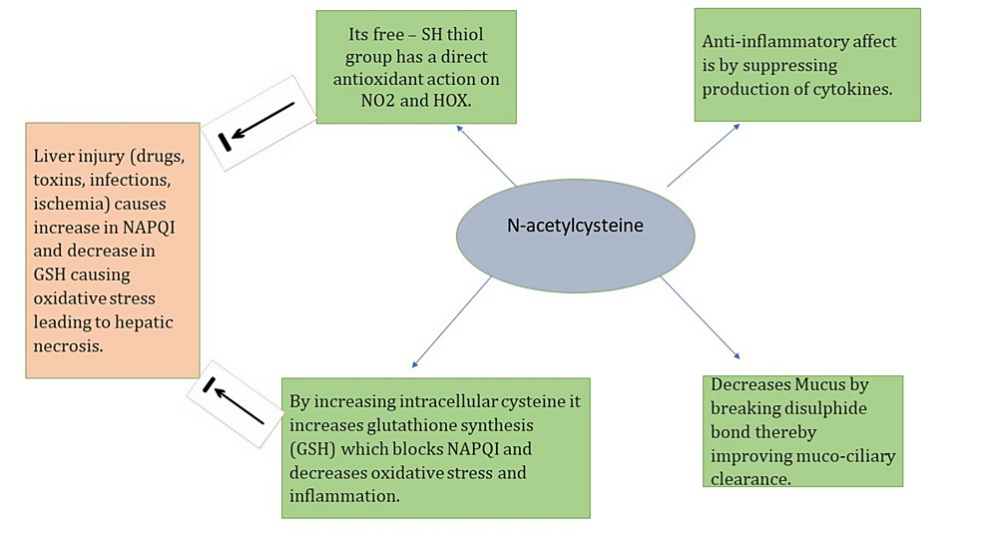
Mechanism of action for N-Acetylcysteine (NAC). Image credits: Dr. Saleem Sharieff

Based on similar mechanisms (Figure 1), when used early in heterogeneous populations of non-acetaminophen-induced acute liver failure (NAI‑ALF) due to glutathione depletion, including virus-induced acute hepatic failure, mushroom toxin-induced liver failure, acute alcoholic hepatitis, and heat stroke-induced ALF, NAC showed improvement in survival and a decrease in length of hospital stay [17].

In this series of six patients, we found viral hepatitis as a cause of acute liver failure with a common presentation of hepatic encephalopathy. These findings are comparable to other studies from sub-continent in which reported ALF was associated with severe viral hepatitis [10].

Although liver transplant has now become an established treatment option for patients with ALF, but due to a lack of liver transplant facilities, NAC has emerged as a beneficial treatment for non-acetaminophen ALF. Acetaminophen-induced liver failure is more common in the western word, but in south Asia, viral hepatitis is the most common etiology. Most of the published material is on the use of IV NAC, while the oral form of NAC is not widely used in such cases. In Pakistan, IV NAC is not available, so we use oral NAC in our patients. A previous study from Mumtaz et al. [10] also used oral NAC in their patient population with good recovery, as in our study. Although our hospital is a centre for liver transplants, it is still difficult to perform cadaveric transplants in Pakistan at this time, and we do live donor liver transplants with limited resources and also a possibility of facing difficulty in organ availability from family members. In this situation, the use of NAC in acute liver failure is really an excellent option.

We found significant improvement and survival benefit in our patients diagnosed as NAI‑ALF who were treated with NAC, and none of them died or required liver transplantation. Furthermore, the use of NAC was safe and led to a shorter ICU and hospital stay. We did not experience any major side effects from NAC in our patients, and it was well tolerated. Similarly, another study [10] revealed a reduction in NAI‑ALF related mortality with the use of NAC.

Our limitations were the small sample size and the uncommon nature of the disease. However, the duration of our study was also short, i.e., one year. We also did not have a control population as we started NAC in all these six patients while workup was in progress for the possibility of a liver transplant. However, previously, when only supportive treatment was offered [21], the mortality was quite high. With the use of NAC, all of our patients recovered without requiring a liver transplant.

## Conclusions

Acute liver failure is a life-threatening condition with a high mortality rate but is potentially reversible. Although liver transplant is a treatment of choice, it is unfortunately not readily available. We suggest that the use of N-acetyl-l-cysteine (NAC) along with conventional treatments will benefit patients with NAI‑ALF in centers where transplant facilities are not available and even in patients with liver transplant facilities while undergoing work-up for transplant. It not only improves survival but also decreases the length of hospital stays.

## References

[1] Stravitz RT, Lee WM (2019). Acute liver failure. Lancet.

[2] Bernal W, Lee WM, Wendon J, Larsen FS, Williams R (2015). Acute liver failure: A curable disease by 2024?. J Hepatol.

[3] Bernal W, Wendon J (2013). Acute liver failure. N Engl J Med.

[4] Ostapowicz G, Fontana RJ, Schiødt FV (2002). Results of a prospective study of acute liver failure at 17 tertiary care centers in the United States. Ann Intern Med.

[5] Nabi T, Dar SA, Ra N (2020). Effect of N-acetylcysteine in indeterminate acute liver failure. Int J Cur Res Rev.

[6] Acharya SK, Dasarathy S, Kumer TL (1996). Fulminant hepatitis in tropical population: clinical course, cause, and early predictors of outcome. Hepatology.

[7] Davidson DG, Eastham WN (1966). Acute liver necrosis following overdose of paracetamol. Br Med J.

[8] Prescott LF, Newton RW, Swainson CP (1974). Successful treatment of severe paracetamol overdosage with cysteamine. Lancet.

[9] Smilkstein MJ, Bronstein AC, Linden C (1991). Acetaminophen overdose: A 48-hour intravenous N-acetylcysteine treatment protocol. Ann Emerg Med.

[10] Mumtaz K, Azam Z, Hamid S, Abid S, Memon S, Ali Shah H, Jafri W (2009). Role of N-acetylcysteine in adults with non-acetaminophen-induced acute liver failure in a center without the facility of liver transplantation. Hepatol Int.

[11] Chughlay MF, Kramer N, Spearman CW, Werfalli M, Cohen K (2016). N-acetylcysteine for non-paracetamol drug-induced liver injury: a systematic review. Br J Clin Pharmacol.

[12] Ezeriņa D, Takano Y, Hanaoka K, Urano Y, Dick TP (2018). N-acetyl cysteine functions as a fast-acting antioxidant by triggering intracellular H2S and sulfane sulfur production. Cell Chem Biol.

[13] Spapen H, Zhang H, Demanet C, Vleminckx W, Vincent JL, Huyghens L (1998). Does N-acetyl-L-cysteine influence cytokine response during early human septic shock?. Chest.

[14] Lee WM, Stravitz RT, Larson AM (2012). Introduction to the revised American Association for the Study of Liver Diseases Position Paper on acute liver failure 2011. Hepatology.

[15] Walayat S, Shoaib H, Asghar M, Kim M, Dhillon S (2021). Role of N-acetylcysteine in non-acetaminophen-related acute liver failure: an updated meta-analysis and systematic review. Ann Gastroenterol.

[16] Katoonizadeh A, Decaestecker J, Wilmer A (2007). MELD score to predict outcome in adult patients with non-acetaminophen-induced acute liver failure. Liver Int.

[17] Lee WM, Hynan LS, Rossaro L (2009). Intravenous N-acetylcysteine improves transplant-free survival in early stage non-acetaminophen acute liver failure. Gastroenterology.

[18] Morabito V, Adebayo D (2014). Fulminant hepatitis: Definitions, causes and management. Health.

[19] Darweesh SK, Ibrahim MF, El-Tahawy MA (2017). Effect of N-Acetylcysteine on mortality and liver transplantation rate in non-acetaminophen-induced acute liver failure: a multicenter study. Clin Drug Investig.

[20] Nabi T, Nabi S, Rafiq N, Shah A (2017). Role of N-acetylcysteine treatment in non-acetaminophen-induced acute liver failure: A prospective study. Saudi J Gastroenterol.

[21] Khuroo MS, Kamili S (2003). Aetiology and prognostic factors in acute liver failure in India. J Viral Hepat.

[22] O’Grady JG, Alexander GJ, Hayllar KM, Williams R (1989). Early indicators of prognosis in fulminant hepatic failure. Gastroenterology.

[23] Mormone E, George J, Nieto N (2011). Molecular pathogenesis of hepatic fibrosis and current therapeutic approaches. Chem Biol Interact.

[24] Diesen DL, Kuo PC (2011). Nitric oxide and redox regulation in the liver: part II. Redox biology in pathologic hepatocytes and implications for intervention. J Surg Res.

[25] Edwards L, Wanless IR (2013). Mechanisms of liver involvement in systemic disease. Best Pract Res Clin Gastroenterol.

[26] Li S, Tan HY, Wang N, Zhang ZJ, Lao L, Wong CW, Feng Y (2015). The role of oxidative stress and antioxidants in liver diseases. Int J Mol Sci.

